# Phenolic Profiles in Olive Leaves from Different Cultivars in Tuscany and Their Use as a Marker of Varietal and Geographical Origin on a Small Scale

**DOI:** 10.3390/molecules29153617

**Published:** 2024-07-31

**Authors:** Francesca Borghini, Gabriella Tamasi, Steven Arthur Loiselle, Michele Baglioni, Stefano Ferrari, Flavia Bisozzi, Sara Costantini, Cristiana Tozzi, Angelo Riccaboni, Claudio Rossi

**Affiliations:** 1 Department of Biotechnology, Chemistry and Pharmacy, University of Siena, Via Aldo Moro 2, 53100 Siena, Italy; gabriella.tamasi@unisi.it (G.T.); steven.loiselle@unisi.it (S.A.L.); michele.baglioni@unisi.it (M.B.); flavia.bisozzi@student.unisi.it (F.B.); sara.costantini@student.unisi.it (S.C.); claudio.rossi@unisi.it (C.R.); 2 Santa Chiara Lab, University of Siena, Via Valdimontone 1, 53100 Siena, Italy; cristiana.tozzi@unisi.it (C.T.); angelo.riccaboni@unisi.it (A.R.); 3 Centre for Colloid and Surface Science (CSGI), University of Florence, Via della Lastruccia 3, 50019 Sesto Fiorentino, Italy; 4 ISVEA, Istituto per Lo Sviluppo Viticolo Enologico ed Agroindustriale, Via Basilicata 1-5, Località Fosci, 53036 Poggibonsi, Italy; s.ferrari@isvea.it; 5 Department of Business and Law, University of Siena, Piazza San Francesco 8, 53100 Siena, Italy

**Keywords:** discriminant factorial analysis, geographical origin markers, olive leaves, phenolic profile, Tuscany

## Abstract

Olive leaves are a rich source of polyphenols with healthful properties and represent one of the most abundant waste products of olive oil production. The aims of this study were to explore the phenolic composition of olive leaves from the three main Tuscan cultivars (Leccino, Moraiolo and Frantoio) collected in Siena and Grosseto provinces and to investigate the possible use of these compounds as varietal and geographic origin markers. Discriminant factorial analysis (DFA) was used for distinguishing between different cultivars and locations. Apigenin and caffeoyl-secologanoside showed significant differences between cultivars. DFA showed that ligstroside, apigenin and luteolin have the most influence in determining the differences between sites, whereas total polyphenols, olacein and hydroxytyrosol acetate allowed for separation between leaves from the same province. The results of the present study indicate that concentrations of phenolic compounds, measured through high-resolution mass spectrometry, can be used as a marker for both the cultivar and of geographical origin of olive leaves, and possibly of olive-related products, as well as across small geographic scales (less than 50 km distance between sites).

## 1. Introduction

Olive leaves represent a rich source of polyphenols, especially secoiridoids (oleuropein, verbascoside), and flavonoids (luteolin-7-*O*-glucoside, rutin and apigenin-7-*O*-glucoside), but also hydroxytyrosol and simple phenols, which are responsible for the health benefits of olive leaf extract (OLE) [1]. OLE has been shown to have antioxidant, anti-inflammatory, hypoglycemic, neuroprotective and antimicrobial properties [2,3,4,5,6,7,8,9]. At the same time, olive leaves represent a major and underutilized waste product of the olive oil industry, constituting 10% of the entire olive harvest weight [10,11], making their disposal an economic and environmental problem while representing a potentially valuable by-product [12,13]. Olive leaves have several applications in a range of sectors, such as cattle feed, energy generation, fertilizers, novel materials and food and pharmaceutical products [14]. In the perspective of the circular economy, there is a clear need to develop processes that use these underutilized by-products for productive purposes [15], for example, the extraction of both sugars and antioxidants [16]. There are clear opportunities for OLE addition to food productions, increasing their antioxidant, microbiological and nutritional properties [10,17]. A recent work [18] evaluated how the antioxidant and anti-inflammatory properties of olive oil could be increased by the addition of OLE, with a new sustainable approach to valorize olive leaves. The addition of dry ground olive leaves prior to malaxation process significantly increases the polyphenolic content and antioxidant capacity of virgin oil [19]. Likewise, lyophilized leaf extracts were microencapsulated to employ phenolic compounds and minerals of olive leaves in functionalized yogurt [7]. In addition, olive leaf extracts have been used for other applications, such as cosmetic formulations [20] and commercial dietary supplements [21]. In this context, the detailed study of phenolic profiles of different varieties of olive leaves from different regions in the world recently gathered some interest in the scientific community.

The phenolic profile of olive leaves is affected both by biotic and abiotic factors such as cultivar, leaf age, moisture content, and geographical origin, as well as the sampling time and/or extraction processes [22,23,24]. Studies have shown that the phenolic profile of olive fruits and oil is influenced by environmental factors, such as water stress [25] and climatic conditions [26,27], and is likely to have a similar impact on leaves. Generally, an increased water stress implies a rise in phenolic content in fruit and oil [25,28]. Likewise, environmental stress can also modify the metabolism of polyphenols in leaves: a study conducted on Meski cultivar leaves sampled in three different climatic zones in Tunisia detected an increase in phenolic compounds, tannins, phytosterols, carotenoids and flavonoids with a concomitant decrease in chlorophyll in leaves cultivated in arid areas [29]. Several authors [30] demonstrated that the total phenolic content (TPC) of olive cultivars from six sites in Anatolia (Turkey) decreases in a directly proportional way to the geographical altitude. A recent study on leaves of the Chetoui cultivar showed that not only pedological features but also environmental conditions influence their phenolic profile [31], and, specifically, leaves from higher altitude locations are characterized by high concentrations of secoiridoids, whereas the lower ones are higher in flavonoids. Also, the polyphenolic content of the Chemlali cultivar showed significant variation among samples from nine Tunisian regions [32]. The phenolic profile of olive leaves can also be used to help identify the geographical origin of olive-based products [30,33,34].

Consequently, several multivariate approaches were proposed by different authors to study the phenolic composition of olive leaves both in relation to the genotype and environmental factors, i.e., geographical location and pedological variables, that may also influence the nutritional potential of olive leaves. Multivariate analysis is a powerful statistical technique used to analyze datasets with multiple variables simultaneously. As previously reported, it allows for the exploration of complex relationships between variables and for understanding the underlying structure of the data [35,36,37,38,39]. For example, the phenolic profiles and antioxidant activities of the leaf extracts of nine olive genotypes were determined and analyzed using a hierarchical cluster analysis, which allowed for the division of the genotypes into three clusters [40]. 

Phenolic compounds are mostly affected by the variety, whereas several mineral nutrients’ (Mg and Fe) content is mostly useful for differentiating locations, and some secoiridoids (verbascoside and hydroxytyrosol) are also affected by the cultivation and adopted agronomic methodologies (conventional or ecological) [41]. Also, Lukić et al. [42] found polyphenols more useful to differentiate olive leaves cultivars, while Zn and P were found to be more useful for differentiating the locations. Recently, the principal component analysis (PCA) of untargeted metabolic profiling of twelve olive leaf cultivars showed good segregation among cultivars [43]. 

The discrimination of 13 varieties of olive trees from the same geographic area and the differentiation of six geographical zones of the same variety (Arbequina) was achieved using the phenolic compounds in leaves as variables [44]. Different multivariate analyses were applied (PCA, hierarchical cluster analysis, soft independent modeling of class analogy) and the most significant variables on the PCA models were the content in oleuropein, verbascoside, apigenin-7-glucoside and luteolin-7-glucoside.

In this context, there is room for improving the methods currently used to assess this approach in different relevant world areas, where the production of olive oil, and, subsequently, the determination of the geographical origin of the products are particularly important. Tuscany is one of the principal producers of extra-virgin olive oil in Italy (with a production of about 25,000 metric tons per year, corresponding to approximately 3% of the total national production), and it has some excellent oils, with protected designation of origin, such as Chianti Classico PDO extra virgin olive oil, Terre di Siena PDO extra virgin olive oil and Tuscan PGI extra virgin olive oil. 

Thus, the aim of this study is to explore the phenolic composition of olive leaves from the three main Tuscan cultivars (Leccino, Moraiolo and Frantoio) from different localities sampled on a relatively small geographical scale (an area of approximately 50 × 100 km^2^) and to evaluate the use of this composition as varietal and geographical markers. Phenolic profiles were determined using liquid chromatography coupled with high-resolution mass spectrometry (HPLC-HRMS) and multivariate analysis was applied to explore the datasets and their relationships. In particular, in this work, the discriminant factorial analysis (DFA) method was used not only to classify samples, but also to identify the most important variables that differ across sampling sites.

## 2. Results

Table 1, Table 2, Table 3 and Table 4 summarize the polyphenol concentrations in olive leaves sampled in the present study, belonging to the Leccino, Moraiolo and Frantoio varieties. Genotype is a major factor of variability in olive leaf phenolic composition [45,46,47]. While a previous study of the same cultivars [48] reports significant variations in hydroxytyrosol and oleuropein, the present one showed that apigenin and caffeoyl-secologanoside provided the best separation between cultivars (Bartlett Test, *p* < 0.01), with higher concentrations of apigenin in Leccino leaves and higher levels of caffeoyl-secologanoside in Frantoio samples compared to the other two. 

Oleuropein-glucoside, hydroxytyrosol acetate and olacein were generally found in the highest concentrations, in agreement with previous studies, i.e., oleuropein was the main component also for nine studied genotypes from Turkey [40,49] and for cultivars from Spain [9,41]. 

The concentrations of flavonoids and secoiridoids of Tuscan samples from this work are comparable with those reported in leaves of six olive cultivars collected at different periods in several Croatian areas [42,50], in samples of Arbequina [51], Manzanilla [24] and other Spanish cultivars [9,41,52]. 

The TPC was higher in the present Tuscany samples (37.3–66.3 mg GAE/g dw) than in leaves from six different sites in Anatolia (Turkey, from 7.3 to 38.7 mg GAE/g dw) [30] or on Tunisian cultivars (23.8–43.1 mg GAE/g dw) [31,34] and Spanish samples (10.38 mg GAE/g dw [52]. The TPC of Tuscan genotypes was comparable to the Manzanilla variety (20–55 mg GAE/g dw) [24], to those reported for Spanish cultivars (52.2–60.6 mg GAE/g dw) [46] and to those of other Italian cultivars (40.9–66.6 mg GAE/g dry leaves), from the same sampling period [48].

**Figure 1:**
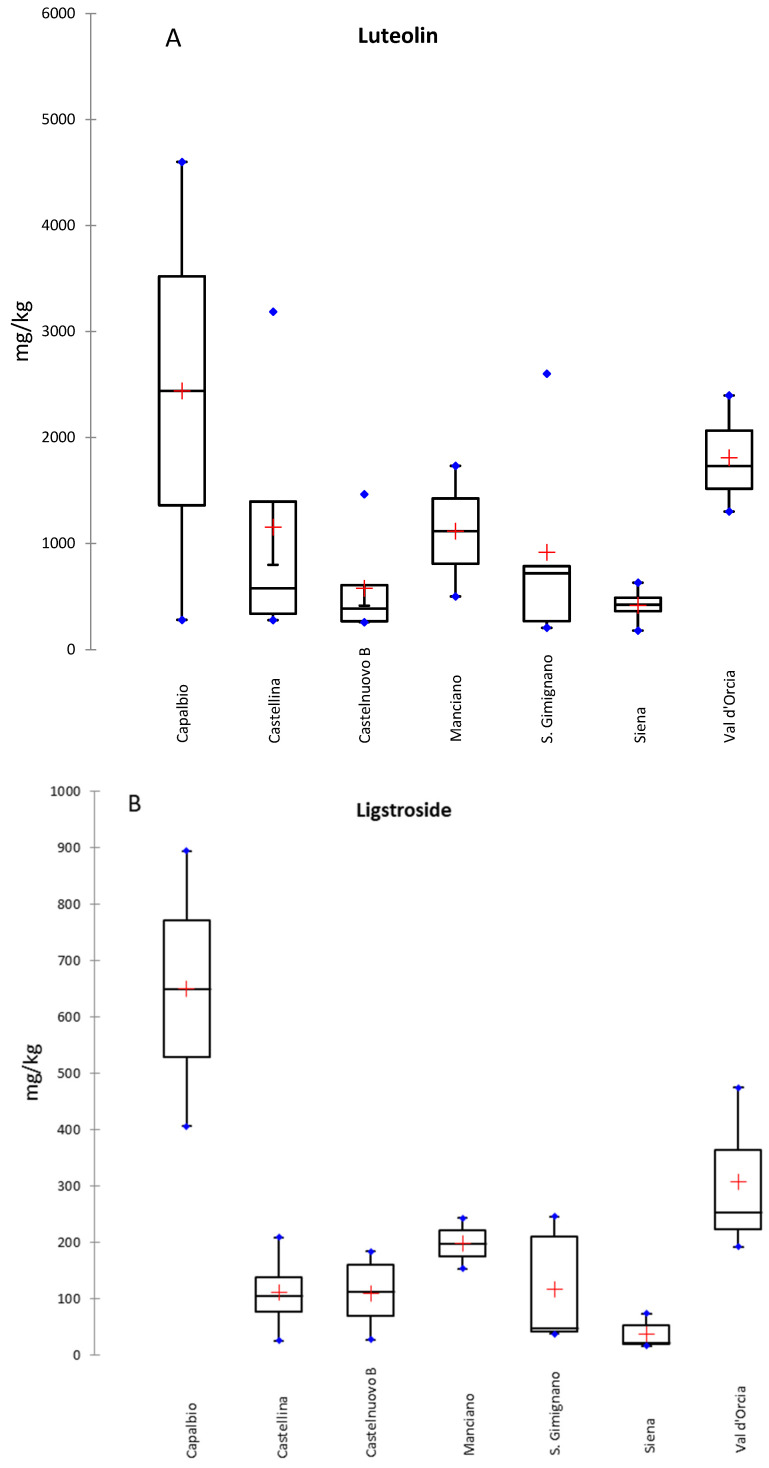
Compounds (concentrations in mg/kg dw) with statistically significant differences between sites (*p* < 0.01, Bartlett test): (**A**) luteolin and (**B**) ligstroside.

Differences between geographic regions were also identified (Bartlett Test, *p* < 0.01); in particular, ligstroside and luteolin were found to be the best markers, particularly high in Capalbio samples (Grosseto province) and in Val d’Orcia, (Siena province, Figure 1). 

As phenol composition and content in both olive fruits and oil have been shown to be influenced by environmental factors, similar drivers may influence leaf conditions. Meteorological data for the summer and autumn of 2022 (http://www.sir.toscana.it) showed hotter and drier conditions for the summer in Capalbio (closer to the sea and at a lower altitude above sea-level, asl) than in Manciano (Grosseto province), even if the annual rainfall was quite comparable (680 mm and 587 mm with 50 and 67 rainy days, respectively, even if 115 mm were recorded all at once on September 4th 2022 in Capalbio). The altitude across the study sites was similar (300 m); thus, it was not possible to associate differences in phenolic composition to altitude.

**Figure 2:**
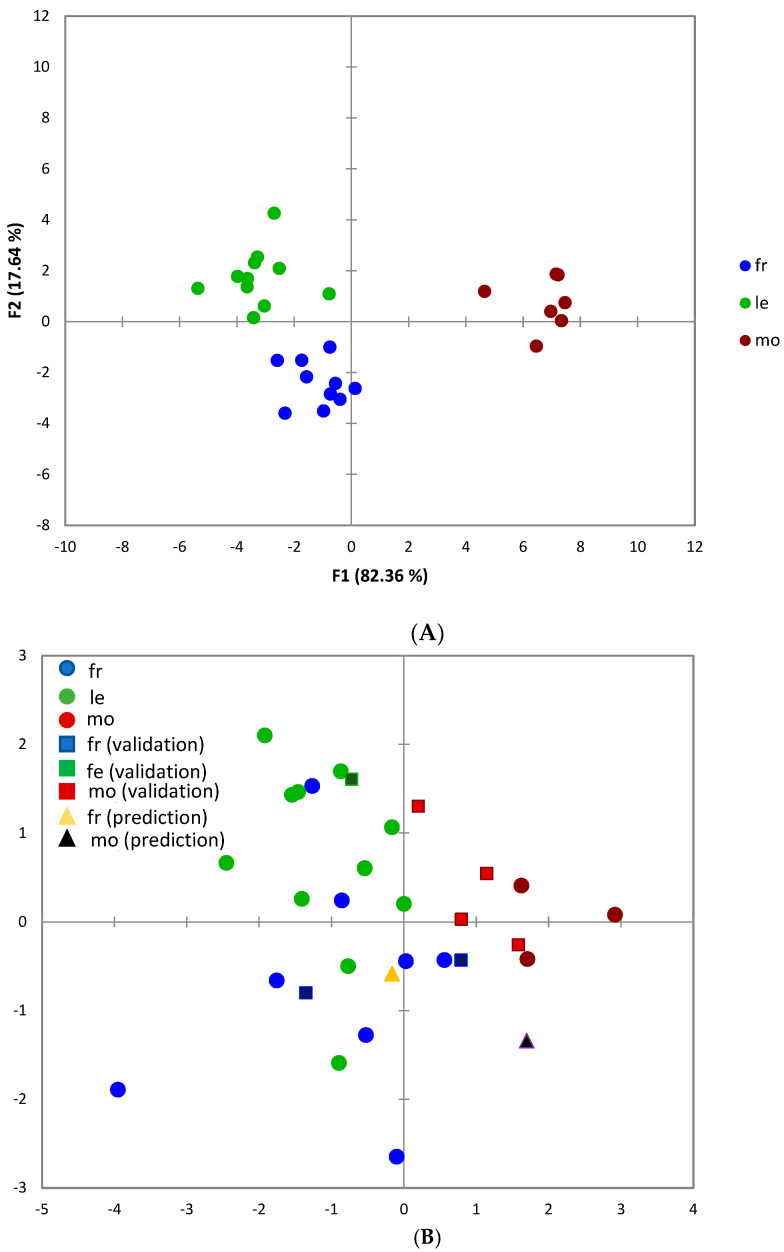
(**A**) Scatter plot of the first and second variables of the DFA in relation to cultivars (Fr = Frantoio, Le = Leccino and Mo = Moraiolo) from Tuscany. (**B**) Scatter plot of the first and second variables of the DFA model constructed using the six selected variables. The circles represent the calibration samples, squares the validation samples; the yellow triangle is the Frantoio from Civitella Marittima and the black triangle is the Canino cultivar sample from Manciano.

Discriminant factorial analysis (DFA) was firstly performed using all variables (n = 29) and then we repeated the analysis by eliminating the multicollinearity highlighted by the analysis of the VIF and the correlation matrix (n = 16). The DFA of phenolic profiles showed a clear separation between cultivars along the two factors (Figure 2A), with Moraiolo separated well on F1, while Frantoio and Leccino separated along F2. In fact, F1, accounting for most of variation (82%), was dominated by flavonoids, luteolin-7-glucoside (correlation 0.407), apigenin (0.368), hydroxytyrosol glucoside (0.350), secoiridoid ligstroside (0.386) and the triterpene maslinic acid. The second factor (F2) was dominated by caffeoyl-secologanoside (0.483) and maslinic acid (−0.404), showing the ability of these compounds to separate more similar cultivars. This pattern is in agreement with previous studies about the genetic assessment of the Tuscan olive germplasm [53].

A DFA model was built based on the most discriminant six variables (see just above) and 22 samples for calibration, whereas the validation was performed using seven randomly selected samples together with a site containing a different cultivar (Canino) and a Frantoio sample from a different site of Grosseto province (Civitella Marittima). The confusion matrices deduced from prior and posterior classifications showed an accuracy of 71% for the seven validation sites. Figure 2B shows that the Frantoio sample from Civitella Marittima was correctly assigned to the Frantoio class, whereas the Canino cultivar does not fall into any of the cultivar groups, as expected.

**Figure 3:**
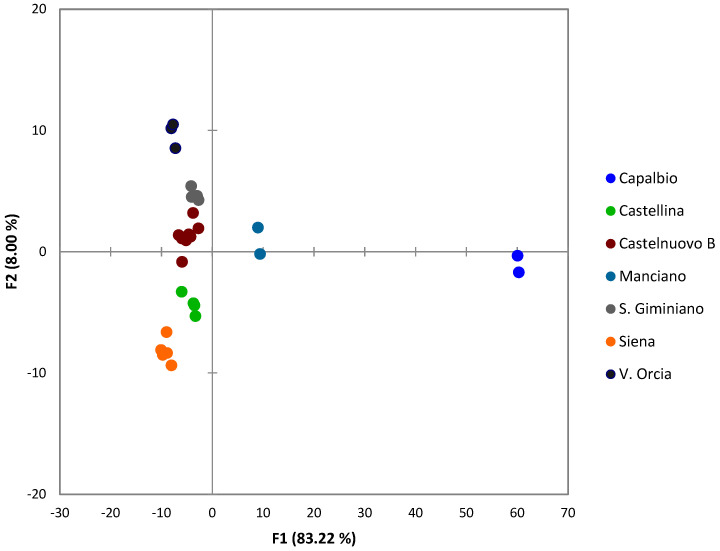
Scatter plot of the first and second variables of the DFA based on the Tuscan leaves sampling sites using sampling sites as dependent variables.

DFA was also used to explore dominant phenols characterizing sampling sites (Figure 3). The first factor (F1) accounted for the majority of the variation between sites (83%), while the second one accounted for 8%. The leaves sampled in Manciano and Capalbio (Grosseto province) show positive values of F1, whereas all those taken in Siena province have negative values. Sites within the same provinces also showed some separation: along F1 for the two Grosseto sites and along F2 for the Siena sites. F1 was dominated by ligstroside (0.753, λ = 0.27), apigenin (0.470, λ = 0.61) and luteolin (0.389, λ = 0.56), while F2 was defined by TPC (0.717, λ = 0.42), olacein (0.566, λ = 0.42) and hydroxytyrosol acetate (0.563, λ = 0.47). These results confirmed the findings of Japón-Lujan et al. [44], identifying apigenin-glucoside and luteolin-glucoside as good geographical markers. Given the limited number of samples from each site, classification accuracy was not determined. 

## 3. Materials and Methods

### 3.1. Chemicals

All reagents (Folin–Ciocalteu’s phenol reagent, gallic acid, hydroxytyrosol, tyrosol, verbascoside, oleuropein, oleanolic acid, oleocanthal, caffeic acid, ferulic acid, coumaric acid, chlorogenic acid, vanillin, rutin, luteolin 7-O-glucoside, luteolin and apigenin) were purchased from Merck (Darmstadt, Germany). All reagents were of analytical grade and distilled water was obtained from a Milli-Q purification system from Millipore (Milford, MA, USA).

### 3.2. Samples and Extraction Protocol

**Figure 4:**
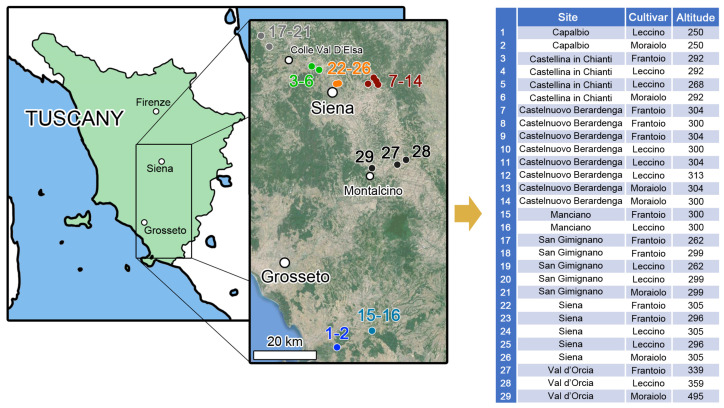
Study area and sampling sites, with relative altitude (m asl) and sampled cultivars.

Olive leaves were collected from 29 farms in Tuscany (central Italy), and most of the samples (n = 25) were from the Siena province, whereas 4 samples were collected from Grosseto province (Figure 4). Different Tuscan olive cultivars (Leccino, Moraiolo and Frantoio) were sampled in the same farm, when available. Leaves were collected from three different trees for each cultivar during olive harvest (September and October 2022) at four cardinal directions along the perimeter of each tree, at operator height, and stored in plastic bag in the dark until arrival at the laboratory. All the samples were carefully washed with ultrapure water, lyophilized (−45 °C, 360 µbar) to constant mass. They were blade-milled (Pulverizette 11, Fritsch) in a liquid nitrogen bath to fine powder (500 µm) before the analysis. The samples were maintained frozen and in dark conditions until analysis. 

The sample extraction was performed according to a slightly modified version of the standard method of the International Olive Council’s methodology for determining phenolic compounds in olive oils (2009), as reported by other authors [52]. Briefly, aliquots of 0.200 g of dry samples were extracted with 2 mL of a methanol/water mixture (80:20 methanol/water) for 15 min at 23 ± 2 °C, in an ultrasonic bath (Argo Lab DU-65, with ultrasonic power = 180 W and frequency = 40 kHz). The extracts were centrifuged (3500 rpm, 15 min), and then the supernatant was filtered with syringe filters (0.22 µm) prior to the HPLC-HRMS analysis. All samples and standards were handled to minimize light exposure and the analyses were performed in triplicate.

### 3.3. Determination of Total Phenol Content (TPC) 

TPC in the leaf extracts was determined by the Folin–Ciocalteu method [5,54] with some modifications: 100 μL of olive extracts or a blank sample (methanol/water, 1:1, *v*/*v*) were diluted with 5 mL of water and then treated with Folin–Ciocalteu reagent (500 μL) and (after 1 min) Na_2_CO_3_ 20% *w*/*v* aqueous solution (1.5 mL). Immediately after gentle shaking, the flasks were made to final volume (10 mL) with water and incubated for 60 min at 25 °C. Absorbance at 760 nm, using a Varian Cary spectrophotometer, was recorded against water. The calibration curves were recorded by using standard solutions of gallic acid in the linear range, 0.25–10.00 mg/L, and R^2^ > 0.990 was accepted for analyses. All samples were analyzed in triplicate. The total phenolic content (TPC) was expressed in gallic acid equivalents (GAE) and per kg of dried sample (dry weight, dw).

### 3.4. HPLC-HRMS Analysis

The content of phenolic and triterpenic compounds was determined by means of UHPLC-HRMS (ultra-high-performance liquid chromatography coupled with high-resolution mass spectrometry). The liquid chromatograph was a Vanquish Flex Thermo Fisher Scientific (Waltham, MA, USA) equipped with a binary pump and a thermostated autosampler. A Hypersil gold column (2.1 × 100 mm 1.7 µm; Thermo Fisher Scientific (Waltham, MA, USA)) was used. The autosampler tray temperature was set at 8 °C, and the column at 40 °C. Gradient elution was performed with water/0.1% formic acid (solvent A) and methanol/0.1% formic acid (solvent B) at a constant flow rate of 300 µL/min; the injection volume was 0.1 µL. An increasing linear gradient of solvent B was used. Separation was carried out in 30 min under the following conditions: 0 min, 5% B; 10 min, 50% B; 12 min, 60% B; 25 min, 90% B; 27 min, 90%; 28 min 5% B and from 28 min to 30, 5% B. An Exploris 120 Orbitrap mass spectrometer (Thermo Fisher Scientific, (Waltham, MA, USA)) equipped with an electrospray ionization (ESI) source in negative mode was used to acquire mass spectra in full mass spectrometry (MS) data-dependent MS2 experiment. The source conditions were the followings: spray voltage (V) 3700, sheath gas 40 arbitrary units (a.u.), auxiliary gas 10 a.u., sheath gas 0 a.u., vaporizer and ion transfer tube temperatures 200 and 325 °C, respectively. The scan range was 90–900 *m*/*z*, with 120,000 Orbitrap resolution in full MS and 15,000 in data-dependent MS^2^. The Thermo Xcalibur 4.5 software was used to control the instrument, whereas Thermo TraceFinder 4.1 was used to quantify the compounds using external standard curve method in the linear range 10–1000 µg/L.

Calibration curves with R^2^ > 0.980 were accepted for analyses. Compounds for which commercial standard were not available were quantified as equivalents, using standards from the same family: tyrosol-glucoside as tyrosol equivalent, hydroxytyrosol-glucoside, hydroxytyrosol acetate and secologanoside as hydroxytyrosol equivalent, coumaroyl-secologanoside as coumaric acid equivalent, caffeoyl-secologanoside as caffeic acid equivalent, olacein, hydroxyolacein, ligstroside, hydroxyligstroside and oleoside as oleocanthal equivalent, hydroxyverbascoside as verbascoside equivalent, and maslinic acid as oleanolic acid equivalent. 

The LOD and LOQ were calculated based on the analytical curve by selecting appropriate signal-to-noise ratios (3:1 and 10:1, respectively) and using the standard deviation of the response (σ) and the slope of the curve (S). The LOQ and LOD were 10 and 4 µg/L, respectively.

### 3.5. Statistical Analysis

XLStat for Windows (version 2014.5.03) and Unscrambler X were used for the statistical data elaboration, setting the level of significance at *p* < 0.01. Prior to multivariate analysis, all data were centered to remove or reduce skewness and statistical differences between groups (cultivars, geographic origin) were then defined using ANOVA (Bartlett Test), after verifying that all parameters followed a near-normal distribution. Discriminant factorial analysis (DFA) was applied, i.e., a multivariate statistical technique combining factor analysis (FA) and discriminant analysis (DA). FA was chosen to reduce the dimensionality of the polyphenol profile by extracting a few common factors that explain most of the variance. DA is used to classify a set of observations into two or more groups based on the resulting factors, using their linear combination as discriminant functions. The homogeneity of variances and covariances (DA) was confirmed by Box tests, using a *p*-value > 0.001. Outliers were identified using Mahalanobis distance while the degree of multicollinearity was determined using variance inflation factor (VIF). 

Olive cultivars (n = 3) and sampling sites were used separately as qualitative dependent (Y) variables. In the case of cultivars, the number of samples was sufficient to also allow for the building of a predictive model, which was subsequently tested, splitting all the samples in calibration (n = 22) and validation (n = 7) sets. 

Wilks’ lambda, λ was used to determine the contribution of each variable to the discriminatory power of the model. The correlation coefficients between each variable and the vector score were used to show the importance of each variable to the group separation.

## 4. Conclusions

The phenolic composition of olive leaves, measured through high-resolution mass spectroscopy, was successfully used to differentiate between cultivars. In fact, using a multivariate DFA approach, it was possible to clearly distinguish between leaves pertaining to Moraiolo, Frantoio and Leccino varieties. In perfect agreement with their “genetic distance”, Moraiolo cluster is well separated from Frantoio and Leccino ones, which are closer. Apigenin and caffeoyl-secologanoside were found to be the best markers for the identification, which can be further improved by including additional phenolic compounds, i.e., luteolin-7-glucoside, hydroxytyrosol glucoside, ligstroside and maslinic acid. A DFA model was then built using these variables, which was cross-validated, yielding a good result of 71% accuracy, and tested—with positive outcome—in the assignment prediction of two additional leaves samples.

On the other hand, ligstroside and luteolin were the most appropriate compounds to assess the geographical origin. DFA further confirmed that a clear clusterization is achieved, indicating that apigenin is also a notable marker to discriminate between samples from the Siena and the Grosseto province, while TPC, together with the hydroxytyrosol acetate and ligstroside, is crucial for the fine separation between clusters of samples pertaining to very close areas from the Siena province.

Further studies with a larger number of participating sites and across multiple harvests could be used to assess pedoclimatic effects on specific phenolic compounds and in relation to different varieties. Recent studies have shown that phenol composition and content in olive leaves, fruits and oil can be significantly affected by environmental factors such as irrigation, harvest date, climatic conditions, tree conditions and management practices with respect to genotype [42]. For example, an increase in water stress determines an increase in phenolic content in olive leaves, fruits and oil, but the response to the environmental stress varies between different varieties.

From the perspective of the circular economy, it would be beneficial to analyze phenol composition in waste leaves present during the pruning period (winter) since studies show that phenolic content changes with season [22].

The results of the present study constitute a milestone to build on as they clearly indicate that phenolic compounds’ concentration, measured through high-resolution mass spectrometry, can be effectively used as a marker both for the cultivar and of geographical origin of olive leaves, and possibly of olive-related products. Targeted metabolomic analysis of phenolic compounds could help define the geo-origin of olive-related products on small geographic scales. Other techniques (e.g., stable isotope fingerprinting, multielement analysis, DNA fingerprinting or a combination of them) are most effective at larger scales (regional or national). This method could be very useful for Italian olive oils, which are high-quality products with some protected designation of origin and a great economic and cultural value. 

## Figures and Tables

**Table 1:** Phenolic alcohols, their derivatives and flavonoids (expressed in g/kg dw, n = 3) and total polyphenol concentration (TPC, expressed in g/kg gallic acid equivalents; g GAE/kg dw, n = 3) in Tuscan olive leaves (Mo = Moraiolo, Le = Leccino, Fr = Frantoio). cv is cultivar.

Site	cv	Hydroxytyrosol	Hydroxytyrosol acetate	Hydroxytyrosol glucoside	Luteolin	Luteolin-7-O-glucoside	Apigenin	Rutin	TPC
Capalbio	Mo	1.5 ± 0.1	36.7 ± 2.5	2.2 ± 0.2	0.28 ± 0.02	1.6 ± 0.1	0.53 ± 0.04	0.46 ± 0.04	37.5 ± 2.5
Capalbio	Le	2.6 ± 0.1	49.8 ± 3.2	14.5 ± 0.8	4.6 ± 0.3	4.3 ± 0.3	7.95 ± 0.75	0.56 ± 0.09	42.6 ± 3.2
Castellina	Mo	0.56 ± 0.08	37.1 ± 3.1	0.16 ± 0.013	3.2 ± 0.2	2.2 ± 0.2	0.97 ± 0.09	0.31 ± 0.02	42.1 ± 3.9
Castellina	Le	1.8 ± 0.1	5.1 ± 0.2	11.0 ± 0.6	0.28 ± 0.02	6.3 ± 0.6	0.45 ± 0.09	0.65 ± 0.05	44.2 ± 2.9
Castellina	Le	0.61 ± 0.05	16.6 ± 1.2	3. 7 ± 0.2	0.80 ± 0.05	5.6 ± 0.5	0.67 ± 0.07	0.47 ± 0.04	49.7 ± 3.6
Castellina	Fr	0.79 ± 0.05	10.5 ± 0.6	5.8 ± 0.3	0.36 ± 0.02	6.3 ± 0.9	0.53 ± 0.08	0.45 ± 0.04	41.8 ± 3.3
Castelnuovo B.	Le	3.0 ± 0.1	5.1 ± 0.2	24.7 ± 1.6	0.27 ± 0.02	7.2 ± 0.7	0.40 ± 0.08	0.98 ± 0.15	60.2 ± 5.2
Castelnuovo B.	Fr	0.44 ± 0.08	35.2 ± 1.6	3.2 ± 0.2	1.19 ± 0.07	4.1 ± 0.4	0.55 ± 0.09	0.49 ± 0.09	40.6 ± 3.8
Castelnuovo B.	Le	1.8 ± 0.1	39.5 ± 2.8	18.2 ± 0.9	1.47 ± 0.08	6.6 ± 0.7	3.32 ± 0.41	0.77 ± 0.14	60.5 ± 4.6
Castelnuovo B.	Mo	0.69 ± 0.08	14.3 ± 0.8	7.8 ± 0.5	0.26 ± 0.02	4.4 ± 0.3	0.08 ± 0.01	0.52 ± 0.10	58.8 ± 4.6
Castelnuovo B.	Fr	1.6 ± 0.1	8.3 ± 0.6	12.4 ± 0.7	0.26 ± 0.02	6.7 ± 0.5	0.14 ± 0.03	0.47 ± 0.08	57.3 ± 4.9
Castelnuovo B.	Le	3.1 ± 0.1	25.8 ± 1.8	22.7 ± 0.7	0.40 ± 0.03	8.2 ± 0.8	1.48 ± 0.21	0.88 ± 0.11	55.8 ± 3.9
Castelnuovo B.	Mo	1.1 ± 0.1	13.9 ± 1.1	10.9 ± 0.7	0.41 ± 0.03	5.6 ± 0.5	0.13 ± 0.03	0.67 ± 0.09	53.2 ± 4.2
Castelnuovo B.	Fr	1.9 ± 0.1	11.6 ± 0.9	14.8 ± 0.9	0.38 ± 0.02	11.9 ± 0.8	0.19 ± 0.03	0.88 ± 0.11	55.1 ± 4.3
Manciano	Fr	0.68 ± 0.08	49.6 ± 2.6	5.9 ± 0.3	1.73 ± 0.12	7.7 ± 0.7	0.74 ± 0.09	0.49 ± 0.09	52.5 ± 3.8
Manciano	Le	1.32 ± 0.05	10.0 ± 0.7	12.1 ± 0.9	0.50 ± 0.03	3.4 ± 0.3	0.81 ± 0.09	0.55 ± 0.11	46.2 ± 3.6
San Gimignano	Mo	1.75 ± 0.05	22.8 ± 1.0	14.4 ± 1.0	0.72 ± 0.06	8.8 ± 0.8	0.17 ± 0.03	1.18 ± 0.12	66.3 ± 5.2
San Gimignano	Fr	0.93 ± 0.06	6.6 ± 0.4	6.9 ± 0.3	0.21 ± 0.02	6.0 ± 0.6	0.12 ± 0.02	0.68 ± 0.11	58.3 ± 2.9
San Gimignano	Fr	1.7 ± 0.1	80.6 ± 5.6	8.9 ± 0.6	2.60 ± 0.22	5.0 ± 0.4	3.85 ± 0.35	1.47 ± 0.14	72.4 ± 4.1
San Gimignano	Le	1.4 ± 0.1	33.6 ± 2.3	18.6 ± 1.2	0.79 ± 0.06	9.5 ± 0.9	3.69 ± 0.55	0.78 ± 0.12	57.6 ± 3.9
San Gimignano	Le	1.3 ± 0.1	7.3 ± 0.4	12.6 ± 0.8	0.27 ± 0.02	5.3 ± 0.5	0.30 ± 0.06	0.61 ± 0.11	42.5 ± 2.9
Siena	Mo	2.7 ± 0.1	10.1 ± 0.6	26.6 ± 1.3	0.49 ± 0.04	5.8 ± 0. 5	0.41 ± 0.08	0.83 ± 0.11	54.8 ± 3.2
Siena	Fr	0.79 ± 0.05	6.2 ± 0.4	4.5 ± 0.2	0.63 ± 0.05	7.2 ± 0.6	1.33 ± 0.24	0.71 ± 0.12	35.7 ± 1.9
Siena	Le	1.07 ± 0.06	13.1 ± 0.7	6.5 ± 0.4	0.42 ± 0.05	6.4 ± 0.6	0.24 ± 0.02	0.44 ± 0.09	51.6 ± 2.6
Siena	Le	2.0 ± 0.1	18. 6 ± 1.0	17.7 ± 1.0	0.36 ± 0.04	5.2 ± 0.5	0.24 ± 0.01	0.70 ± 0.11	59.5 ± 4.1
Siena	Fr	3.8 ± 0.2	3.7 ± 0.3	21.3 ± 1.3	0.18 ± 0.02	3.2 ± 0.3	0.25 ± 0.02	0.53 ± 0.08	41.8 ± 3.6
Val d’Orcia	Le	1.8 ± 0.1	25.5 ± 1.8	17.7 ± 1.0	2.40 ± 0.21	6.4 ± 0.5	3.76 ± 0.33	0.56 ± 0.09	51.8 ± 3.2
Val d’Orcia	Fr	0.74 ± 0.09	72.3 ± 5.2	5.8 ± 0.5	1.30 ± 0.11	7.7 ± 0.5	0.88 ± 0.15	0.63 ± 0.09	56.8 ± 2.9
Val d’Orcia	Mo	0.97 ± 0.05	78.2 ± 5.4	0.29 ± 0.05	1.73 ± 0.11	0.17 ± 0.02	0.70 ± 0.12	0.26 ± 0.05	37.3 ± 1.5

**Table 2:** Secoiridoids (expressed in g/kg dw, n = 3) in Tuscan olive leaves (Mo = Moraiolo, Le = Leccino, Fr = Frantoio). OLE oleuropein, OLE agly oleuropein aglycone, OLC oleocanthal, LIGS ligstroside, LIGS agly ligstroside aglycone.

Site	Cultivar	OLE	OLE agly	OLC	LIGS	LIGS agly	Olacein	Oleoside
Capalbio	Mo	12.0 ± 0.9	5.7 ± 0.5	0.47 ± 0.05	3.5 ± 0.3	0.89 ± 0.05	40.0 ± 2.8	4.3 ± 0.4
Capalbio	Le	14. 9 ± 1.5	5.5 ± 0.8	0.85 ± 0.13	3.1 ± 0.3	0.41 ± 0.08	54.1 ± 3.8	2.2 ± 0.3
Castellina	Mo	6. 8 ± 0.7	3.8 ± 0.5	0.80 ± 0.09	3.1 ± 0.3	0.21 ± 0.04	40.6 ± 3.2	2.4 ± 0.4
Castellina	Le	15.3 ± 1.2	7.2 ± 0.8	0.09 ± 0.02	4.3 ± 0.5	0.02 ± 0.003	9.7 ± 0.9	8.2 ± 1.1
Castellina	Lle	24.0 ± 1.8	3.4 ± 0.6	0.33 ± 0.05	8.2 ± 0.8	0.11 ± 0.02	18.5 ± 1.6	7. 4 ± 0.8
Castellina	Fr	26.2 ± 2.2	2.6 ± 0.4	0.25 ± 0.05	6.8 ± 1.0	0.09 ± 0.02	11.4 ± 1.5	3.7 ± 0.6
Castelnuovo B.	Le	35.6 ± 2.9	2.5 ± 0.3	0.43 ± 0.06	9.0 ± 0.8	0.05 ± 0.01	9.2 ± 1.2	9.3 ± 1.2
Castelnuovo B.	Fr	15.1 ± 1.8	3.3 ± 0.4	1.07 ± 0.15	4.4 ± 0.5	0.13 ± 0.02	38.9 ± 2.9	3.6 ± 0.7
Castelnuovo B.	Le	23.0 ± 3.2	7.5 ± 1.1	0.85 ± 0.11	7.3 ± 0.8	0.18 ± 0.03	43.6 ± 4.2	5.5 ± 0.7
Castelnuovo B.	Mo	38.5 ± 2.5	4.6 ± 0.7	0.45 ± 0.06	6.7 ± 1.0	0.16 ± 0.03	15.9 ± 2.1	11.1 ± 1.6
Castelnuovo B.	Fr	44.2 ± 3.9	2.6 ± 0.3	0.32 ± 0.06	10.7 ± 1.	0.09 ± 0.02	9.4 ± 1.1	7.7 ± 1.0
Castelnuovo B.	Le	12.6 ± 0.9	9.7 ± 1.1	0.50 ± 0.07	3.4 ± 0.5	0.16 ± 0.03	27.5 ± 1.9	3.3 ± 0.4
Castelnuovo B.	Mo	44.4 ± 3.9	4.9 ± 0.7	0.58 ± 0.08	8.9 ± 0.9	0.08 ± 0.01	15.0 ± 1.5	15.3 ± 2.1
Castelnuovo B.	Fr	36.8 ± 3.3	3.7 ± 0.6	0.45 ± 0.06	8.1 ± 1.1	0.03 ± 0.01	13.0 ± 1.8	6.6 ± 1.0
Manciano	Fr	18.9 ± 1.7	6.8 ± 1.0	0.78 ± 0.09	6.2 ± 0.9	0.24 ± 0.04	54.2 ± 4.8	2.2 ± 0.4
Manciano	Le	28.8 ± 2.1	2.9 ± 0.5	0.22 ± 0.04	7.9 ± 1.1	0.15 ± 0.03	11.0 ± 1.1	5.5 ± 0.8
San Gimignano	Mo	23.4 ± 2.4	6.2 ± 0.9	0.60 ± 0.09	5.7 ± 0.8	0.21 ± 0.04	25.5 ± 1.9	4.3 ± 0.5
San Gimignano	Fr	42.6 ± 3.8	3.4 ± 0.6	0.29 ± 0.05	11.8 ± 1.5	0.04 ± 0.01	8.0 ± 1.0	11.7 ± 1.1
San Gimignano	Fr	4.7 ± 0.7	9.4 ± 1.1	1.90 ± 0.25	1.7 ± 0.3	0.25 ± 0.04	86.8 ± 7.9	0.6 ± 0.1
San Gimignano	Le	34.2 ± 2.9	4.6 ± 0.6	2.48 ± 0.33	11.0 ± 1.3	0.04 ± 0.01	37.8 ± 5.2	8.2 ± 1.2
San Gimignano	Le	31.5 ± 2.8	5.4 ± 0.7	0.13 ± 0.03	7.7 ± 1.1	0.05 ± 0.01	8.2 ± 1.1	7.3 ± 1.0
Siena	Mo	36.1 ± 2.8	2.2 ± 0.4	0.78 ± 0.11	10.1 ± 1.2	0.02 ± 0.004	10.8 ± 1.3	4.3 ± 0.6
Siena	Fr	7.4 ± 1.1	1.7 ± 0.2	0.53 ± 0.08	3.8 ± 0.6	0.02 ± 0.004	7.8 ± 1.2	2.6 ± 0.4
Siena	Le	26.5 ± 1.9	3.1 ± 0.6	0.52 ± 0.07	9.2 ± 1.1	0.07 ± 0.01	14.8 ± 0.9	7.6 ± 1.1
Siena	Le	34.9 ± 2.5	3.2 ± 0.5	0.86 ± 0.12	10.5 ± 1.2	0.05 ± 0.01	20.5 ± 1.9	5.9 ± 0.8
Siena	Fr	16.0 ± 1.5	2.4 ± 0.4	0.05 ± 0.01	4.1 ± 0.6	0.02 ± 0.004	5.7 ± 0.8	3.7 ± 0.7
Val d’Orcia	Le	38.0 ± 2.8	3.7 ± 0.7	0.57 ± 0.09	7.6 ± 1.0	0.19 ± 0.03	27.9 ± 3.5	20.7 ± 2.8
Val d’Orcia	Fr	28.6 ± 2.5	7.4 ± 1.0	0.93 ± 0.11	8.9 ± 1.3	0.25 ± 0.05	78.1 ± 6.6	4.8 ± 0.7
Val d’ Orcia	Mo	7.5 ± 1.1	3. 7 ± 0.5	0.79 ± 0.11	1.6 ± 0.3	0.47 ± 0.07	85.9 ± 7.2	2.4 ± 0.4

**Table 3:** Secoiridoids (expressed in mg/kg dw, n = 3) in Tuscan olive leaves (Mo = Moraiolo, Le = Leccino, Fr = Frantoio).

Site	Cultivar	Nuzheide	Secologanoside	Caffeoyl- secologanoside	Coumaroyl- secologanoside
Capalbio	Mo	0.08 ± 0.01	0.08 ± 0.01	0.05 ± 0.01	10.8 ± 1.4
Capalbio	Le	0.05 ± 0.01	0.10 ± 0.01	0.05 ± 0.01	0.05 ± 0.01
Castellina	Mo	0.07 ± 0.01	0.14 ± 0.02	0.04 ± 0.01	0.05 ± 0.01
Castellina	Le	0.05 ± 0.01	0.57 ± 0.09	0.05 ± 0.01	0.05 ± 0.01
Castellina	Le	0.06 ± 0.01	0.31 ± 0.05	7.7 ± 1.1	21.7 ± 2.4
Castellina	Fr	0.05 ± 0.01	0.31 ± 0.04	0.05 ± 0.01	0.05 ± 0.01
Castelnuovo B.	Le	42.6 ± 3.9	0.52 ± 0.08	0.05 ± 0.01	0.05 ± 0.01
Castelnuovo B.	Fr	36.8 ± 5.1	0.13 ± 0.02	0.05 ± 0.01	2.0 ± 0.3
Castelnuovo B.	Le	46.3 ± 3.9	0.33 ± 0.04	0.06 ± 0.01	0.05 ± 0.01
Castelnuovo B.	Mo	97.8 ± 5.1	0.39 ± 0.05	0.05 ± 0.01	3.7 ± 0.5
Castelnuovo B.	Fr	32.5 ± 2.5	0.65 ± 0.09	8.0 ± 1.2	15.1 ± 1.2
Castelnuovo B.	Le	21.6 ± 1.9	0.32 ± 0.05	0.05 ± 0.01	0.05 ± 0.01
Castelnuovo B.	Mo	149.3 ± 11.9	0.57 ± 0.09	0.05 ± 0.01	0.88 ± 0.12
Castelnuovo B.	Fr	52.2 ± 4.8	0.53 ± 0.09	13.0 ± 0.9	17.8 ± 1.9
Manciano	Fr	63.0 ± 5.2	0.12 ± 0.02	0.05 ± 0.01	3.02 ± 0.36
Manciano	Lle	58.4 ± 4.5	0.38 ± 0.06	0.05 ± 0.01	0.05 ± 0.01
San Gimignano	Mo	0.10 ± 0.01	0.39 ± 0.04	0.05 ± 0.01	24.8 ± 2.9
San Gimignano	Fr	0.05 ± 0.01	0.57 ± 0.07	7.5 ± 1.0	12.0 ± 1.3
San Gimignano	Fr	0.08 ± 0.01	0.28 ± 0.05	3.6 ± 0.6	47.0 ± 3.9
San Gimignano	Le	56.5 ± 3.8	0.33 ± 0.06	4.5 ± 0.7	10.8 ± 1.2
San Gimignano	Le	57.2 ± 4.2	0.33 ± 0.05	0.05 ± 0.01	2.2 ± 0.3
Siena	Mo	133.2 ± 9.6	0.44 ± 0.07	7.9 ± 1.2	48.7 ± 3.9
Siena	Fr	26.4 ± 3.2	0.23 ± 0.04	0.05 ± 0.01	0.05 ± 0.01
Siena	Le	38.7 ± 2.8	0.37 ± 0.07	0.05 ± 0.01	6.6 ± 0.8
Siena	Le	140.1 ± 8.5	0.38 ± 0.07	0.06 ± 0.01	34.5 ± 2.7
Siena	Fr	23.6 ± 1.9	0.38 ± 0.07	0.05 ± 0.01	0.05 ± 0.01
Val d’Orcia	Le	0.08 ± 0.02	0.35 ± 0.07	0.05 ± 0.01	7.0 ± 0.8
Val d’Orcia	Fr	0.07 ± 0.01	0.29 ± 0.05	19.3 ± 1.5	47.6 ± 3.9
Val d’Orcia	Mo	0.05 ± 0.01	0.05 ± 0.01	0.10 ± 0.01	0.05 ± 0.01

**Table 4:** Phenolic acids and triterpenes (expressed in mg/kg dw, n = 3) in Tuscan olive leaves (Mo = Moraiolo, Le = Leccino, Fr = Frantoio).

Site	Cultivar	Elenoic acid	Verbascoside	Maslinic acid	Oleanolic acid
Capalbio Grosseto	Mo	0.29 ± 0.05	1109 ± 112	779 ± 94	822 ± 99
Capalbio Grosseto	Le	5.6 ± 0.7	389 ± 43	846 ± 105	938 ± 113
Castellina	Mo	14.9 ± 1.4	4.0 ± 0.6	1062 ± 118	1077 ± 130
Castellina	Le	25.2 ± 1.8	37 ± 5	1126 ± 136	1215 ± 158
Castellina	Le	8.7 ± 1.0	25 ± 3	532 ± 64	700 ± 92
Castellina	Fr	7.8 ± 0.9	32 ± 4	448 ± 54	597 ± 78
Castelnuovo B.	Le	0.05 ± 0.01	1522 ± 160	1101 ± 135	1107 ± 144
Castelnuovo B.	Fr	5.6 ± 0.4	81 ± 9	1005 ± 121	983 ± 125
Castelnuovo B.	Le	34.2 ± 2.5	500 ± 57	957 ± 106	1055 ± 127
Castelnuovo B.	Mo	7.0 ± 0.8	323 ± 36	1022 ± 132	1015 ± 132
Castelnuovo B.	Fr	0.05 ± 0.01	216 ± 27	567 ± 72	696 ± 85
Castelnuovo B.	Le	46.8 ± 5.5	1362 ± 150	952 ± 110	1077 ± 115
Castelnuovo B.	Mo	7.2 ± 0.8	198 ± 26	1505 ± 179	1353 ± 158
Castelnuovo B.	Fr	11.9 ± 1.5	410 ± 50	220 ± 29	278 ± 35
Manciano	Fr	10.5 ± 1.4	57 ± 7	1012 ± 128	1080 ± 115
Manciano	Le	0.05 ± 0.01	172 ± 22	661 ± 86	751 ± 85
San Gimignano	Mo	30.3 ± 3.6	433 ± 52	1556 ± 181	1356 ± 142
San Gimignano	Fr	25.0 ± 3.0	283 ± 36	741 ± 89	958 ± 103
San Gimignano	Fr	55.6 ± 6.7	305 ± 37	833 ± 99	950 ± 108
San Gimignano	Le	9.3 ± 1.2	245 ± 34	998 ± 100	1152 ± 125
San Gimignano	Le	9.5 ± 1.3	1598 ± 192	593 ± 77	712 ± 77
Siena	Mo	0.05 ± 0.01	599 ± 180	1283 ± 154	1085 ± 122
Siena	Fr	0.05 ± 0.01	97 ± 14	1381 ± 175	1360 ± 158
Siena	Le	7.5 ± 0.9	110 ± 14	762 ± 92	858 ± 92
Siena	Le	3.0 ± 0.4	283 ± 34	1035 ± 123	994 ± 110
Siena	Fr	7.1 ± 0.9	936 ± 115	607 ± 79	723 ± 82
Val d’Orcia	Le	1.8 ± 0.3	94 ± 13	1204 ± 145	1234 ± 145
Val d’Orcia	Fr	21.2 ± 2.4	242 ± 30	742 ± 96	852 ± 92
Val d’Orcia	Mo	7.2 ± 0.9	63 ± 9	595 ± 16	697 ± 58

## Data Availability

All data for this paper are within the manuscript.
